# Establishment of a cardiac telehealth program to support cardiovascular diagnosis and care in a remote, resource-poor setting in Uganda

**DOI:** 10.1371/journal.pone.0255918

**Published:** 2021-08-06

**Authors:** Alyssa DeWyer, Amy Scheel, Jenipher Kamarembo, Rose Akech, Allan Asiimwe, Andrea Beaton, Bua Bobson, Lesley Canales, Kristen DeStigter, Dhruv S. Kazi, Gene F. Kwan, Chris T. Longenecker, Peter Lwabi, Meghna Murali, Emma Ndagire, Judith Namuyonga, Rachel Sarnacki, Isaac Ssinabulya, Emmy Okello, Twalib Aliku, Craig Sable

**Affiliations:** 1 Virginia Tech Carilion School of Medicine, Roanoke, VA, United States of America; 2 Emory University School of Medicine, Atlanta, GA, United States of America; 3 Gulu Regional Referral Hospital, Gulu, Uganda; 4 Imaging the World, Kampala, Uganda; 5 The Heart Institute, Cincinnati Children’s Hospital Medical Center, Cincinnati, OH, United States of America; 6 The University of Cincinnati School of Medicine, Cincinnati, OH, United States of America; 7 Uganda Heart Institute, Kampala, Uganda; 8 Children’s National Hospital, Washington, DC, United States of America; 9 University of Vermont Medical Center, Burlington, VT, United States of America; 10 Richard A. and Susan F. Smith Center for Outcomes Research in Cardiology, Beth Israel Deaconess Medical Center, Boston, MA, United States of America; 11 Cardiovascular Medicine Section, Department of Medicine, Harvard Medical School, Boston, MA, United States of America; 12 Boston University School of Medicine, Boston, MA, United States of America; 13 Case Western Reserve University School of Medicine, Cleveland, OH, United States of America; 14 University Hospitals Harrington Heart & Vascular Institute, Cleveland, OH, United States of America; 15 George Washington School of Medicine, Washington, DC, United States of America; University of Oklahoma Health Sciences Center, UNITED STATES

## Abstract

**Introduction:**

To address workforce shortages and expand access to care, we developed a telemedicine program incorporating existing infrastructure for delivery of cardiovascular care in Gulu, Northern Uganda. Our study had three objectives: 1) assess feasibility and clinical impact 2) evaluate patient/parent satisfaction and 3) estimate costs.

**Methods:**

All cardiology clinic visits during a two-year study period were included. All patients received an electrocardiogram and echocardiogram performed by a local nurse in Gulu which were stored and transmitted to the Uganda Heart Institute in the capital of Kampala for remote consultation by a cardiologist. Results were relayed to patients/families following cardiologist interpretation. The following telemedicine process was utilized: 1) clinical intake by nurse in Gulu; 2) ECG and echocardiography acquisition in Gulu; 3) echocardiography transmission to the Uganda Heart Institute in Kampala, Uganda; 4) remote telemedicine consultation by cardiologists in Kampala; and 5) communication of results to patients/families in Gulu. Clinical care and technical aspects were tracked. Diagnoses and recommendations were analyzed by age groups (0–5 years, 6–21 years, 22–50 years and > 50 years). A mixed methods approach involving interviews and surveys was used to assess patient satisfaction. Healthcare sector costs of telemedicine-based cardiovascular care were estimated using time-driven activity-based costing.

**Results:**

Normal studies made up 47%, 55%, 76% and 45% of 1,324 patients in the four age groups from youngest to oldest. Valvular heart disease (predominantly rheumatic heart disease) was the most common diagnosis in the older three age groups. Medications were prescribed to 31%, 31%, 24%, and 48% of patients in the four age groups. The median time for consultation was 7 days. A thematic analysis of focus group transcripts displayed an overall acceptance and appreciation for telemedicine, citing cost- and time-saving benefits. The cost of telemedicine was $29.48/visit.

**Conclusions:**

Our data show that transmission and interpretation of echocardiograms from a remote clinic in northern Uganda is feasible, serves a population with a high burden of heart disease, has a significant impact on patient care, is favorably received by patients, and can be delivered at low cost. Further study is needed to better assess the impact relative to existing standards of care and cost effectiveness.

## Introduction

Cardiovascular disease (CVD) remains a leading cause of global mortality [1] and a major contributor to reduced quality of life [2]. CVD accounts for 7–10% of all adult medical admissions to hospitals in Africa, with heart failure contributing 3–7% [3]. Although CVD is experienced at younger ages in low-middle income countries (LMICs) [4], access to echocardiography remains limited, delaying diagnosis and subsequent management [5, 6].

While cardiologists trained in echocardiography provide care in capital cities throughout sub-Saharan Africa, 80% of the population lives in rural settings [7]. Specialists in rural areas are rare, resulting in long waiting times, substantial transportation costs, and high out-of-pocket payments, limiting universal access to care [3, 8, 9]. Decentralization of care through task shifting has demonstrated promise for improving access in this setting [10]. Task shifting can improve care by increasing access, decreasing cost, and freeing higher-level providers to engage in more complex tasks [11]. The combination of task shifting and innovative telemedicine (that can overcome challenges from limited bandwidth), primarily tele-echocardiography, can provide remote populations in LMICs expanded access to medical services [10, 12].

The Uganda Heart Institute (UHI) in Kampala is the sole provider of advanced cardiovascular diagnosis and treatment in Uganda and oversees cardiac care at regional hospitals throughout the country [13]. UHI has prioritized regionalization of care, including electrocardiography (ECG) and echocardiography, to centers in Northern, Western, and Eastern regions of Uganda with the hope of reaching 12–13 sites in the next five years [14]. In Gulu, located six hours north (by road) of Kampala, access to ECG and echocardiography is available at Gulu Regional Referral Hospital (GRRH). Currently, human resources remain a challenge, with cardiologists performing outreach clinic visits for one to two days on a quarterly basis at most. To address the shortage of cardiologists and limited numbers of outreach clinics, we developed a telemedicine program at GRRH, incorporating existing task shifting infrastructure previously established for clinical care and research for rheumatic heart disease (RHD) and heart failure.

Our study had three objectives: 1) assess the feasibility and clinical impact of implementing a telemedicine solution for cardiovascular care delivery, 2) evaluate patient-/parent- satisfaction with telemedicine, and 3) estimate the costs of telemedicine-based regionalization of cardiovascular care in Uganda.

## Methods

### Study setting

This study was conducted between December 1, 2017 and January 31, 2020 at the GRRH inpatient ward and outpatient cardiology clinic, located 332 km from the capital city of Kampala. GRRH is staffed by two medical officers (post graduate residents) and one internal medicine physician in the medical ward, and one medical officer in the children’s ward.

The Research and Ethics Committee at Makerere University School of Medicine and the institutional review board at Children’s National Hospital approved this study. Waiver of consent was used for research objective 1 of this study as it was addressed by review of data from an established clinical program. For objectives 2 and 3, consent and assent were obtained from parents and patients prior to focus groups. All written consent materials were provided in the local language (Acholi) and consent/assent were obtained in the local language as well. Participant information was de-identified during analysis to ensure anonymity and privacy was maintained.

### Clinical telemedicine consultation

The study leveraged existing personnel and operations that were in place at the cardiology clinic at GRRH. This includes two nurses, a research assistant, local physician, and a telemedicine consult coordinator. One nurse (JK) had extensive experience, over five years performing echocardiograms on hand held ultrasound devices. She demonstrated competence in knowledge of RHD, cardiomyopathy, hypertensive heart disease and other common adult cardiac conditions under direct observation by UHI and Children’s National cardiologists. She received an additional week of onsite training by UHI and Children’s National cardiologists in echocardiography assessment of congenital heart disease. An administrative support person in Kampala supported scheduling of UHI cardiologists for consultation. *Imaging the World* provided IT support for implementation of technology in Gulu and Kampala and training of Gulu and UHI staff.

**Fig 1:**
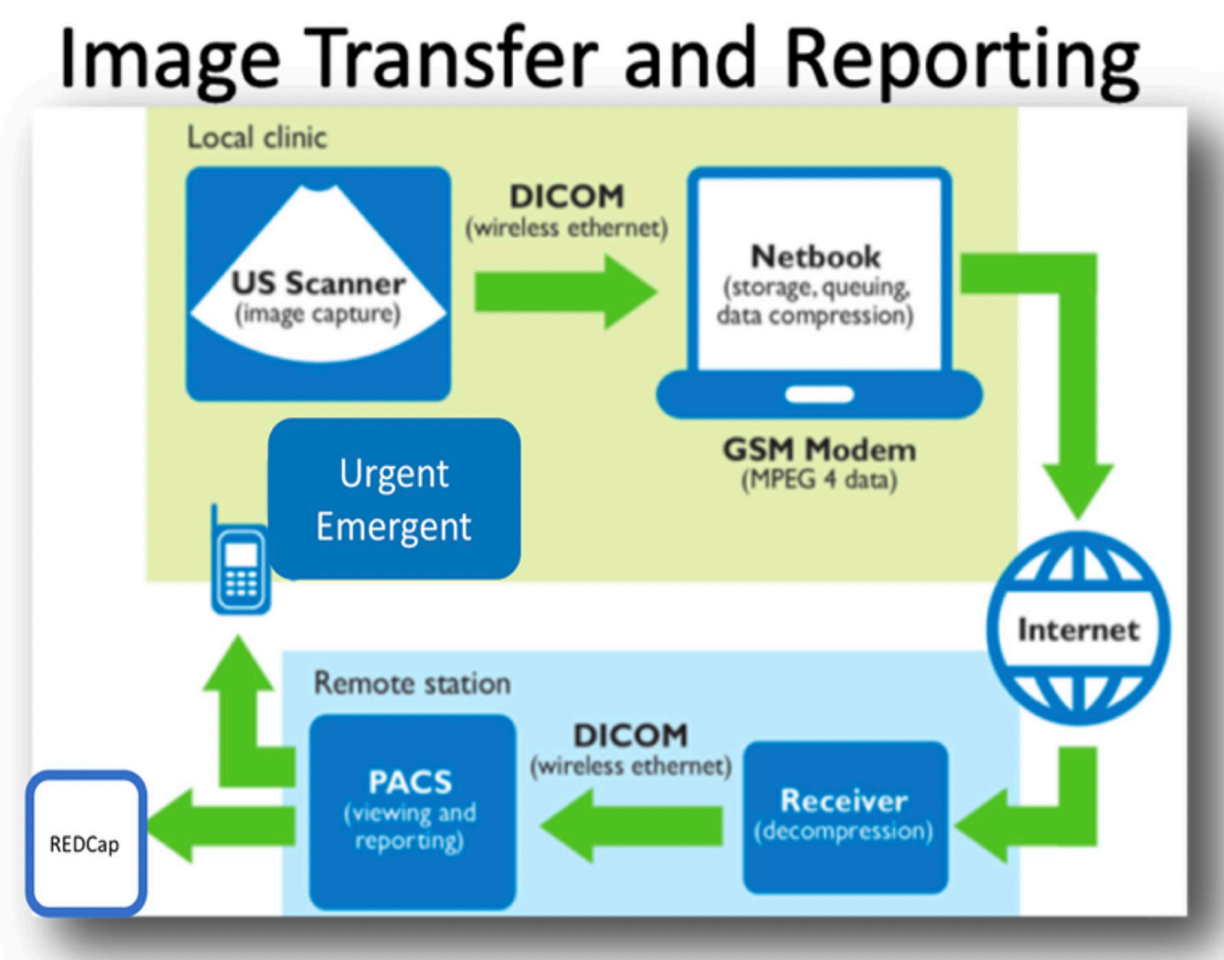
Operational workflow. This figure depicts the operational flow for a patient from intake through final disposition. Steps 1, 2 and 5 take place in Gulu, step 3 takes place in the cloud (data uploaded in Gulu and downloaded in Kampala) and step 4 takes places at Uganda Heart Institute in Kampala. Abbreviations: ECG–electrocardiogram.

**Fig 2:**
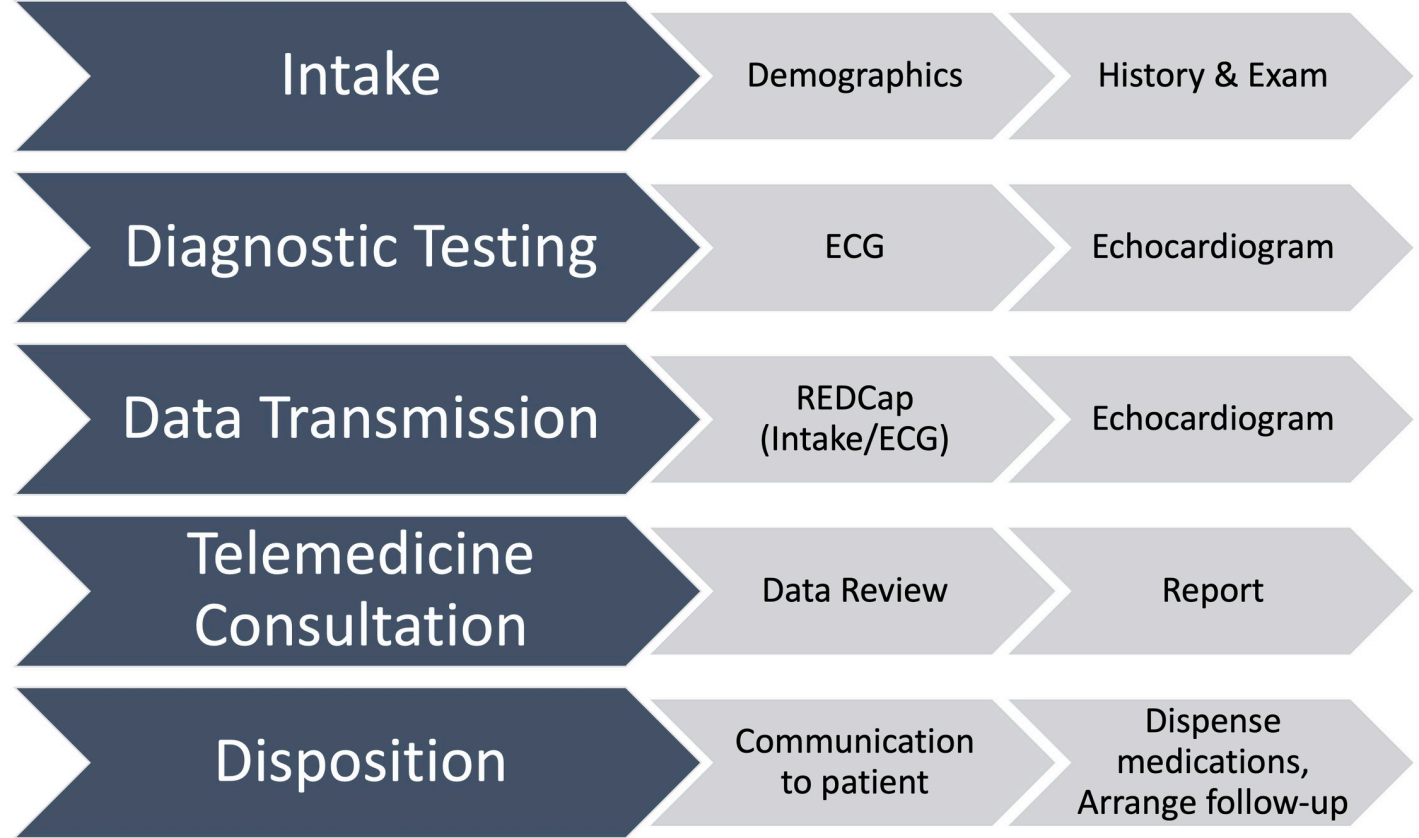
Telemedicine technical flow. This figure depicts the digital flow for the telemedicine process. Abbreviations: DICOM–Digital Imaging and Communications in Medicine; GSM–Global System for Mobile Communications; MPEG–Moving Picture Experts Group; PACS–Picture Archival and Communication System; US–Ultrasound.

All outpatient and inpatient cardiology visits during the study period were included using the following telemedicine process (Fig 1: Operational workflow diagram):

1. **Intake** A GRRH nurse collected type of visit (new or follow-up), patient demographics, chief complaint, past medical history, physical examination findings (performed by nurse), and medications. Additional data collected included time missed from work/school and distance traveled. Intake data including ECGs (smart phone photo) were entered into REDCap [15], a web-based electronic data capture system by nurse and administrative staff at GRRH (S1 File).
2. **Diagnostic testing** ECGs and echocardiograms were performed on all new patients and follow-up patients when recommended on the prior visit
  - **Electrocardiograms** Standard 12 Lead ECGs were recorded (General Electric MAC 5000, Milwaukee, WI, USA) on paper
  - **Echocardiograms** Studies were performed using Philips Lumify (Philips, Bothell, WA, USA), an ultrasound transducer connected by to a Windows-based tablet (Samsung S2 or S4) that operates Lumify software that is publicly available for download at no cost. The tablet is battery powered with an average use time of 4 hours from prior experience. A backup tablet and transducer were available to ensure no downtime. Focused echocardiogram protocols (2D and color Doppler loops and still frames) were utilized. S2 File shows the echocardiography protocol including recommended views for both pediatric and adult studies.
3. **Echocardiography transmission** Echocardiograms were transmitted utilizing an established telemedicine technology solution that is compatible with Lumify provided by *Imaging the World* [16]. We have used this system to successfully compress DICOM echocardiography images from multiple low bandwidth sites in Uganda [12]. Each study was transferred via wireless connection from the echocardiography machine to a notebook computer with proprietary VICAT compression software (Fig 2). The compressed images were automatically uploaded to a Cloud Server (Amazon Web Services) via USB data SIM card. The virtual server was located in Germany with a second physical back up mirrored server in the United States (Data Realm, Phoenix, AZ, USA). Images were available to UHI cardiologists for viewing and making measurements using a PACS workstation (McKesson, Vancouver, British Columbia). UHI providers were required to access images via the server in Germany.
4. **Telemedicine consultation** At the end of each visit, the staff at GRRH communicated with the consult coordinator at UHI that a telemedicine consultation had been performed via closed loop SMS or WhatsApp message for elective consultations. Telemedicine consultation was conducted through asynchronous review of REDCap database (clinical data and ECG) and echocardiograms (McKesson). Five staff cardiologists (EO, IS, TA, PL, JN) from UHI supervised consultation services with additional support from UHI cardiology fellows. The goal for routine consultations was to have a final report completed by a UHI cardiologist within one week. Direct communication with the cardiologist took place for urgent inpatient consultations.
5. **Patient/Parent communication** The nurse at GRRH communicated preliminary results to patients and families immediately after the visit and updated them with final treatment and follow-up recommendations after the final consultation was entered into REDCap by UHI cardiologists. A local physician at GRRH wrote medicine prescriptions that were recommended by UHI cardiologists. The telemedicine process did not include direct verbal communication between UHI physicians and patients/parents in Gulu.

For clinical data analysis, patients were divided into four age groups: 0–5, 6–21, 22–50, and >50 years old. Diagnoses, medical management, patient disposition, distance traveled, and time missed from work/school were compared between groups. Additional diagnostic details were described for patients with congenital and valvular heart disease. Comparisons between age groups were made using chi-square or Fisher exact test for categorical variables and Wilcoxon-Mann-Whitney test for continuous variables; statistical significance was defined as a p value < 0.05. Turnaround times and technical issues including upload and download times were tracked throughout the study.

### Patient/parent satisfaction

A mixed methods approach involving interviews and surveys was used to collect data on patient perception. Staff members trained in qualitative research methods first conducted three focus groups with eight to twelve patients and patient parents to collect narratives on the patient experience. The 40 individuals who were purposively invited to participate in the group were identified by the GRRH clinic staff based on heterogeneity (age group) and their average or above-average responsiveness to the clinic’s reminder calls. The focus group discussion guide, which consisted of eight thematic questions and fourteen suggested follow-up questions was developed by the investigator team to investigate acceptability of the telemedicine intervention [17]. The research staff who conducted the focus groups in the field reviewed and modified the guide with the investigators to assure cultural appropriateness and promote a conversational tone. Focus group moderators were invited to modify the discussion guide as unanticipated themes emerged in the conversation. Audio recordings of the focus groups conducted in the participants’ primary language (Acholi) were professionally translated and transcribed into English prior to analysis. In addition, a 20-question survey modified from published telemedicine quantitative research instruments [17] was used to assess participants’ perceptions of telemedicine and measure preference for telemedicine when compared with regular in-person clinic visits. Surveys were administrated verbally by the research team and responses were collected on paper then entered into REDCap.

The focus group transcripts were coded and analyzed using qualitative description research methods [18]. Four members of the research team who did not conduct the focus groups independently analyzed the transcripts with inductive coding methods in Microsoft Word. Team members independently created codes for the first transcript and identified recurrent patterns and themes. The team convened to compare and combine their codes and themes into an iterative codebook, which was used as a guide in coding subsequent transcripts. When team members disagreed on a proposed code, the group discussed the code until reaching consensus to either include or remove codes from the master codebook. As the master codebook was refined, the team clustered related codes into hierarchical frames and themes. We used descriptive statistics to summarize demographic data. Frequency calculations were utilized in surveys that evaluated patients’ preferences to telemedicine when compared with regular clinic visits.

### Cost analysis

We estimated the healthcare sector costs of telemedicine-based cardiovascular care. Costs borne by the healthcare system were categorized into those related to the facility, equipment, disposables, and personnel. The costs for this analysis were based on building telemedicine on the foundation of preexisting infrastructure and a nurse competent in performing echocardiography. The costs of developing an outpatient clinic and training a sonographer are not included in this analysis. We estimated costs in Uganda Schillings and converted to US dollars using 2019 conversion rate (3,500 Uganda Schilling = 1 US dollar) [19]. Equipment costs incorporated straight-line depreciation over the estimated lifetime of the equipment. Personnel costs were estimated using the time-driven activity-based costing (TDABC) approach [20], based on direct observation and reporting by program staff. Costs borne by patients were estimated from surveys and focus groups as part of objective 2. Our analysis of telemedicine costs excluded the cost of services that existed prior to telemedicine, i.e., those necessary for the general operations of the cardiology clinic regardless of whether telemedicine was available.

## Results

### Telemedicine clinical care and feasibility

A total of 1,324 patients utilized telemedicine services during the 2-year study period; including 140 children between 0 and 5 years old, 424 patients between 6 and 21 years old, 360 patients between 22 and 50, and 400 patients over the age of 50. The vast majority of visits, 1,285 (97%) were new patient visits. There were 33 urgent, inpatient consultations. There were a higher proportion of females in patients over 21 years old (61%) than in patients 21 years old and younger (52%, p < 0.001).

Diagnoses by age group are shown in Table 1. Normal studies made up 47%, 55%, 76% and 45% of patients in the four age groups from youngest to oldest (p < 0.0001 comparing 22–50 group to each other group, other comparisons NS). Congenital heart disease was the most common diagnosis in the under 5 group and valvular heart disease (predominantly RHD) was the most common diagnosis in the older three age groups. Hypertensive heart disease and cardiomyopathy were also common in the oldest age group. The most common congenital heart defects were ventricular septal defect (n = 20), atrial septal defect (n = 16), atrioventricular septal defect (n = 12), patent ductus (n = 9), complex congenital defects (n = 8, including double outlet right ventricle, Ebstein anomaly, truncus arteriosus, single ventricle) and tetralogy of Fallot (n = 6). The vast majority 123/155 (79%) of patients 50 years and younger with valvular heart disease had RHD. Conversely, only 7/73 (9.6%) patients over age 50 with valvular heart disease had RHD. Specific RHD diagnoses included latent RHD (n = 19), mild RHD (n = 26), moderate RHD (n = 25), severe RHD (n = 58) and repaired RHD/valve replacement (n = 2). Of those with mild, moderate, or severe RHD, 51 had isolated mitral regurgitation, 12 had isolated mitral stenosis, 7 had isolated aortic insufficiency and 39 had mixed valve disease.

**Table 1:** Diagnoses.

Diagnosis	0 to 5 years (n = 140)	6 to 21 years (n = 424)	22–50 years (n = 360)	Over 50 (N = 400)
Normal*	66 (47%)	233 (55%)	275 (76%)^1^	181 (45%)
Congenital Heart Disease	65 (46%)	56 (13%)	2 (0.6%)	1 (0.3%)
Valve/Rheumatic Heart Disease	4 (2.9%)	123 (29%)	28 (7.8%)	73 (18%)
Hypertensive Heart Disease	0	4 (0.9%)	17 (4.7%)	58 (15%)
Dilated /Ischemic Cardiomyopathy	3 (2.1%)	4 (0.9%)	21 (5.8%)	56 (14%)
Pericardial Disease	0	3 (0.7%)	7 (1.9%)	5 (1.3%)
Right Heart Disease	2 (1.4%)	1 (0.2%	8 (2.2%)	17 (4.3%)
Arrythmia	0	0	2 (0.6%)	8 (2.0%)
Aortic aneurysm	0	0	0	1 (0.3%)

^1^p < 0.0001 comparing 22–50 age group to each other group for normal diagnosis.

Recommendations for medication and follow-up are shown in Table 2. Medications were prescribed to 31%, 31%, 24%, and 48% of patients in the four age groups (p < 0.001 when comparing oldest age group to all other groups, other comparisons NS). Heart failure medications were the most common in all age groups, with diuretics and angiotensin converting enzyme inhibitors prescribed most frequently. Penicillin prophylaxis was the second most commonly prescribed medication in the 6-21-year age group and anti-hypertensive medications were second most common in the older age group. Youngest and oldest patients were less likely to be discharged from follow-up: 44%, 49%, 70% and 40% in the 4 age groups respectively (p < 0.0001 when comparing 22–50 age group to all other groups, p = 0.01 when comparing 6–21 age group to oldest age group, other comparisons NS). The youngest age group had the highest rate of transfer to tertiary care center (p < 0.001 when compared to each of the other groups).

**Table 2:** Medications and disposition.

Medication	0 to 5 years (n = 140)	6 to 21 years (n = 424)	22–50 years (n = 360)	Over 50 (N = 400)
None	97 (69%)	291 (69%)	275 (76%)	206 (52%)^1^
Penicillin	1 (0.7%)	76 (18%)	6 (1.7%)	2 (0.5%)
Heart Failure	41 (29%)	96 (23%)	44 (12%)	115 (29%)
NSAID	1 (0.7%)	7 (1.7%)	7 (1.9%)	30 (7.5%)
Anti-hypertensive		2 (0.5%)	41 (11%)	76 (19%)
Anti-arrhythmic			11 (3.1%)	5 (1.3%)
Lipid lowering			1 (0.3%)	5 (1.3%)
Anti-coagulation			8 (2.2%)	23 (5.8%)
**Disposition**				
Discharge	61 (44%)^3^	208 (49%)	252 (70%)^2^	160 (40%)^3^
Outpatient follow-up	47 (34%)	164 (39%)	56 (16%)	167 (42%)
Telemedicine follow-up	6 (4.3%)	39 (9.2%)	26 (7.2%)	36 (9%)
Admission to regional hospital	6 (4.3%)	9 (2.1%)	17 (4.7%)	25 (6.3%)
Transfer to national tertiary hospital	20 (14%)^4^	4 (0.9%)	9 (2.5%)	12 (3.0%)

^1^p < 0.001 comparing over 50 age group to each other group for medication recommendation.

^2^p < 0.0001 comparing 22–50 age group to each other group for discharge recommendation.

^3^p = 0.01 comparing 6–21 age group to over 50 age group for discharge recommendation.

^4^p < 0.001 comparing 0–5 age group to each other group for transfer to national hospital.

There was a skewed distribution of travel distance, with the average (37 km) being significantly higher than the median distance traveled (18 km). Families of patients in the youngest age group traveled the farthest (median 32 km, IQR 58 km), followed by older children (median 19.5 km, IQR 29 km), older adults (median 19.5 km, IQR 41 km, and younger adults (median 6 km, IQR 42 km). All comparisons except older children vs. older adults were statistically significant. Only 29% (388/1324) of all patients/families reported taking time off of work or school for the appointment: 6%, 34%, and 20%, and 36% respectively across the 4 age groups.

There were no technical failures due to interruption in upload service from Gulu or malfunctioning telemedicine software or hardware. One of the echocardiography transducers had to be replaced in month 13 of the study, but this did not result in any interruption in performance of echocardiograms. There was a 5 week down-time period in Kampala due to hardware malfunction in month 15 of the study. Adult studies contained between 15–20 loops and 3–4 still frames with files sizes ranging from 80–100 MB. Pediatric studies contained 18–25 loops and 4–6 still frames with files sizes ranging from 100–125 MB. Upload times per study session (all studies done in a given day) ranged from 60 minutes to 6 hours and took place overnight. If upload was interrupted due to temporary internet network outages, the upload would resume from where it was interrupted when internet access was restored. Download times in Kampala averaged 20–30 minutes per study in the first half of study period and 5 to 10 minutes in the second year when internet speed improved (change in internet service provider). Studies were downloaded by a consult coordinator in Kampala. Once downloaded, the physicians were able to view studies seamlessly, however they could only view one study at a time. Physicians spent approximately 30 minutes per consultation including reviewing echocardiograms, reviewing REDCap information and completing consultation reports in REDCap. The median turn-around time was 7 days (IQR 13 days) with 23% of studies interpreted within 48 hours, 54% interpreted within 7 days and 70% interpreted within 10 days. The skewed distribution of turnaround time was related to downtime and recovery periods.

### Patient/parent satisfaction

Surveys were administered to 26 parent/guardians and 34 adult patients, and 30 participants (out of 40 who were invited) engaged in three different focus groups (8–12 per group). All participants completed the surveys; one participant left one question blank. Table 3 reflects response rates to a question directly about participants’ perceptions of telemedicine and Table 4 reflects response rates to questions comparing telemedicine to standard care (after encounter was completed). Detailed responses to focus groups with thematic coding are shared in S3 File.

**Table 3:** Attitudes about telemedicine consultation.

	Yes	No	Unsure
Comfortable with telemedicine?	59 (98.3%)	1(1.7%)	0 (0%)
Telemedicine improved access to care?	57 (95.5%)	1 (1.7%)	2 (3.3%)
Understood diagnosis given by telemedicine?	56 (93.3%)	3 (5.0%)	1 (1.7%)
Understood treatment recommended by telemedicine?	49 (81.7%)	8 (13.3%)	3 (5.0%)
Questions answered by clinician during visit?	55 (91.8%)	3 (5%)	2 (3.3%)
Seen more quickly through telemedicine	56 (93.3%)	4 (6.7%)	0 (0%)
Results delivered quickly through telemedicine?	53 (88.3%)	6 (10.0%)	1 (1.7%)
Shorter distance traveled with telemedicine visit?	57 (95.5%)	2 (3.3%)	1 (1.7%)
Missed less time from school/work with telemedicine?	56 (93.3%)	2 (3.3%)	2 (3.3%)
Spent less money on visit with telemedicine?	56 (93.3%)	3 (5.0%)	1 (1.7%)

**Table 4:** Preferences for type of care delivery.

	Telemedicine visit at GRRH	Physician visit at GRRH	No difference	Unsure
Easier to schedule an appointment	55 (91.7%)	1 (1.7%)	1 (1.7%)	3 (5.0%)
Shorter waiting time	42 (70.0%)	4 (6.7%)	9 (15.0%)	5 (8.3%)
Appointment time more convenient	56 (93.3%)	0 (0%)	2 (3.3%)	2 (3.3%)
Less time being uncertain about diagnosis	53 (88.3%)	1 (1.7%)	4 (6.7%)	2 (3.3%)
Results available more quickly	53 (88.3%)	1 (1.7%)	5 (8.3%)	1 (1.7%)
Which do you spend less time travelling for appointments	48 (80%)	10 (16.7%)	1 (1.7%)	1 (1.7%)
Stay closer to come	56 (93.3%)	2 (3.3%)	2 (3.3%)	0 (0%)
Miss less school/work	40 (66.7%)	13 (21.7%)	6 (10%)	1 (1.7%)
Spend less on food & travel	50 (83.3%)	1 (1.7%)	9 (15.0%)	0 (0%)
More anxiety surrounding the appointment	26 (43.3%)	8 (13.3%)	14 (23.3%)	11 (18.3%)

#### Benefits and acceptance of telemedicine

A thematic analysis of focus group transcripts displayed an overall acceptance and appreciation for telemedicine, citing cost- and time-saving benefits, with some participants encouraging the expansion of telemedicine to rural communities. As described by one participant, *“if I were to go to Mulago it would be too costly in terms of transport and other additional costs*. *As peasant farmers we barely have [the] money they may need and sometimes it may lead to death*”. Results from the surveys support major themes identified from the focus groups. 98.3% of respondents felt comfortable with telemedicine. Compared to where they would traditionally receive a cardiology consultation or check-up, 95.5% felt they had traveled a shorter distance, 93.3% felt they had missed less time from school/work and spent less money on their health visit and 88.3% felt their results were available more quickly. When compared with regular clinic visits, 93.3% of survey respondents reported having more convenient appointment times and 88.3% reported spending less time being uncertain about their diagnoses. Overall, 95.5% felt telemedicine improved their access to care.

#### Concerns with telemedicine

While the surveys and focus groups displayed an overall acceptance and appreciation for telemedicine, participants did voice reservations around this new platform of healthcare. Although 93.3% of survey respondents reported understanding their diagnosis, only 81.2% reported understanding their suggested treatment plan. Themes also emerged surrounding participants’ reported concerns with a lack of physician contact and overall trust in the telemedicine system, with one participant stating, *“One bad thing about telemedicine is that*, *you do not have a physical interaction with your doctor to explain more of what you feel or how you feel*. *And secondly using telemedicine may [cause one to] lose hope since you are not seeing the doctor directly so how can [you] believe it…”* Participants also reported technical limitations and uncertainty with application with regard to emergency care.

### Cost analysis

Table 5 shows the annual costs of each item and estimates of the percent of costs utilized by the telemedicine (as an increment from prior clinical operations) are provided. Based on an annual telemedicine volume of 720 visits per year utilized in this study, the cost of telemedicine was $29.48/visit. Some of the costs are fixed (cloud hosting per study and percentage of Kampala cardiologist time) while others would decrease (in terms of cost/per study) with increased utilization. It is estimated that the capacity listed below could accommodate up to 200 visits per month or 1,920 annual visits. If this optimal capacity were utilized, the cost/visit would decrease to approximately $16.00 per patient visit. Separate from telemedicine costs, the charge for ECG and echocardiogram at GRRH was $5.40 and $8.20 USD respectively and mean medication costs were $2.00. Average personal out-of-pocket expenses for transportation, meals and accommodation were $4.42, $0.26 and $0.07 respectively. Only 20 patients reported any costs related to lodging.

**Table 5:** Costs of telemedicine care delivery (based on 720 annual visits per year).

Amortized Telemedicine Equipment costs*	**Cost**	**Life Span (years)**	Annual cost*	**% for telemed**	**Cost/Visit**
Echo Probes (2)	$16,000	6	$2,667	75%	$2.78
Echo tablets (2)	$1,000	3	$333	100%	$0.46
VICAT Notebook (2)	$1,600	3	$533	100%	$0.74
**Recurring Telemedicine Technology Costs**	**Cost/month**	**Months**	**Annual cost**	**% for telemed**	**Cost/Visit**
Airtime	$50	12	$600	75%	$0.63
Cloud hosting: Subscription	$44	12	$528	100%	$0.73
Cloud hosting: Per study fee					$1.00
**Telemedicine Technology Support Staff**	**Cost/hour**	**Hours/month**	**Annual cost**	**% for telemed**	**Cost/Visit**
Technical support person: Uganda	$12	12	$1,728	100%	$2.40
Technical admin person: Uganda	$12	4	$576	100%	$0.80
Technical team travel			$600	100%	$0.83
**Local Clinic Costs**	**Cost/month**	**Months**	**Annual cost**	**% for telemed**	**Cost/Visit**
Office Supplies	$150	12	$1,800	25%	$0.63
Utilities	$100	12	$1,200	25%	$0.42
Rent	$1,000	12	$12,000	10%	$1.67
**Staff**	** **	** **	**Annual Salary**	**% for telemed**	**Cost/visit**
Echo Nurse			$9,640	25%	$3.35
Clinic Nurse			$5,200	25%	$1.81
Gulu consult coordinator/Admin Support			$3,480	50%	$2.42
Gulu House Officer			$12,000	5%	$0.83
Kampala consult coordinator/Admin Support			$6,960	25%	$2.42
Kampala Cardiologist (N = 5)			$32,000 x 5	2.5%	$5.56
**Total Cost/Patient Visit (720 annual visits)**		** **	** **	** **	**$29.48**

*Assuming straight-line depreciation of capital expenses over the life-time of the equipment.

## Discussion

This paper is the first to describe the clinical characteristics, technical implementation, patient/parent satisfaction and cost of telecardiology in a low-income African country. In this setting, we show that telecardiology feasible, serves a population with a high burden of heart disease, has a significant impact on patient care, is favorably received by patients and parents, and can be delivered at low cost. Nearly half of all patients in our study had abnormal findings, with an even higher rate among children 5 years and younger and those over 50. Additionally, telemedicine excluded disease for a significant number of patients avoiding unnecessary treatment, stress, and travel. One third of patients received at least one medication and subsequent follow-up was recommended in more than half of all patients. RHD was one of the most common diagnoses in our cohort and likely accounts for the preponderance of females in the middle age groups. The potential for telemedicine to positively impact the lives of young adult women with heart disease is a significant benefit of this program [21]. Focus group and survey findings suggest that the majority of patients and families would have experienced major financial hardship in traveling to the capital if telemedicine had not been available, resulting in delayed diagnosis and treatment.

The management of cardiovascular disease requires access to specialists for accurate diagnosis and timely initiation of therapy. Cardiologists trained in echocardiography have made diagnosis available at referral centers in capital cities throughout sub-Saharan Africa [7]. However, available healthcare human resources are limited and unequal physician distribution exacerbates the mismatch between supply and demand: 80% of physicians live in urban areas, whereas 80% of the population is rural [22]. Decentralization of care holds promise for improving access. Our findings apply telemedicine to successful strategies previously used in other East African Countries. In Rwanda, a strategy employing portable echocardiography and simplified algorithms for diagnosis and initial management of heart failure has been developed and utilized in several district hospitals since 2006 [5]. Evaluation of the program structure concluded that training nurses in simplified protocols and basic echocardiography is a potentially successful approach to decentralized care for this vulnerable population.

Our clinical findings highlight the case burden of cardiovascular disease seen in a referral clinic in Sub-Saharan Africa. Hypertensive disease, RHD, and dilated cardiomyopathies account for over 75% of cases of heart failure in Africa [9, 23]. Congenital heart defects are also an important cause of morbidity and mortality in the young, especially in children under 5 years of age [24, 25]. Over 40 million people worldwide are living with RHD and RHD is responsible for over 300,000 deaths and 10 million disability adjusted life years each year [1]. The prevalence remains very high in Sub-Saharan Africa. Between 15 and 40 of 1,000 children have evidence of early RHD when penicillin prophylaxis has the potential to prevent late complications [26, 27]. Unfortunately, many patients present late with heart failure as young adults, secondary to advanced heart valve disease [28, 29]. We and others have shown that task shifting of focused echocardiography to well-trained and supervised non-physician workers with limited focused training is feasible for diagnosis of RHD in Uganda and other LMIC’s [30–32]. Using telemedicine to complement task shifting holds the potential to allow cardiologists in tertiary care centers to provide daily consultative services to patients with cardiovascular disease.

While many publications describe telemedicine use for echocardiography interpretation, education, and training [33–45], none have evaluated its clinical implementation for cardiovascular disease in a low-income country. Telehealth can be a powerful tool to facilitate care of patients with heart disease around the globe. Sharing of images via cloud-based technology can advance research and clinical collaboration for endemic heart disease [30]. More rapid, portable, and innovative uses of tele-echocardiography can provide remote populations in Uganda and other LMICs expanded access to medical services. These include enhanced data compression technology [46], novel training methods for international support [47], and the use of personal devices such as smartphones for near-instantaneous image review [48]. We leveraged technology that is already working throughout Uganda in collaboration with the NGO, *Imaging the World* [16, 49].

Overall, the general acceptance and belief in the telemedicine system suggests ample potential to integrate telemedicine with the current healthcare system. However, some aspects of healthcare remain unchanged by telemedicine, such as patient anxiety while waiting for test results and suboptimal understanding of treatment and diagnosis. In addition, participants often referred to telemedicine as “*the machine*” during focus groups, suggesting that there could be a gap in provider communication and patient understanding. More education (that includes local language and cultural context) on the actual telemedicine system and live virtual interactions with a physician could help bridge this gap.

While some studies have focused on costs of cardiac care delivery in LMIC’s [50–55], there is very little published on evaluating costs of telecardiology in low-resource settings [56]. In the rural Uganda context, the most critical question is assessment of incremental costs of telemedicine compared with the *status quo*, which, in many cases, means limited or no access to cardiovascular specialists. Eberly and colleagues evaluated the costs of integrated, decentralized chronic care for non-communicable diseases in rural Rwanda [57]. Labor, medications, and laboratory testing were the largest components of healthcare costs. Some of per-patient costs would decline with increased utilization of telemedicine. For instance, information technology support would cost less per encounter if the technician time were supporting multiple sites simultaneously.

### Limitations

Even though our study had a significant number of patients, we do not provide comparison between populations served by telemedicine and those served by standard of care. Usual care often requires travel to the national referral hospital for accurate cardiovascular diagnostics and treatment. An assessment of how many patients actually do this was beyond the scope of this study. Further studies are required to compare outcomes in similar populations at risk for heart disease with and without access to telemedicine. Our study utilized a well-trained nurse who had superior echocardiography skills for a wide range of heart disease and was very comfortable with technical aspects of telemedicine. Scaling this program on a national basis would involve a dedicated effort (and incur additional programmatic costs) to train additional staff. Sustainability would require continuous availability of health workers trained in echocardiography. In centers that are understaffed, task shifting and telemedicine may pull needed resources from other activities such as inpatient care, outpatient clinics, and administrative responsibilities. Our feasibility and turn-around data highlight that availability of tertiary care physicians could also be an important rate-limiting step as demand increases.

Our cost analysis is limited only to assessment of costs of telemedicine; more extensive analyses (including long-term savings from averted complications) would be required to comprehensively examine the cost-effectiveness of telemedicine. Cardiovascular disease often affects young individuals in Africa; early diagnosis and treatment may therefore produce long-term gains in quality of life and survival, and reductions in healthcare expenditures. Future economic evaluations of telemedicine must also account for averted productivity losses due to disability or premature mortality, which can be substantial, particularly for conditions like RHD and congenital heart disease that affect children and young adults.

## Conclusions

The large burden of cardiovascular disease in LMIC’s is greatly exacerbated by delayed diagnosis and treatment. Our data show that store and forward transmission of echocardiograms from a remote clinic in northern Uganda for review at a tertiary care center in Kampala (more than 300 km away) is feasible, serves a population with a high burden of heart disease, has a significant impact on patient care, is favorably received by patients and parents, and can be delivered at low cost. Further study is needed to better assess the impact relative to existing standards of care and cost effectiveness. This program has the potential to be deployed on national scale in an affordable manner, potentially resulting in a dramatic impact on outcomes, with high patient satisfaction.

## Supporting information

S1 File Clinical REDCap instruments. (PDF)

S2 File Echocardiography protocols. (DOCX)

S3 File Thematic coded responses to focus group discussions. (DOCX)

S1 Dataset (XLSX)

## References

[1] Roth GA, Mensah GA, Johnson CO, Addolorato G, Ammirati E, Baddour LM, et al. Global Burden of Cardiovascular Diseases and Risk Factors, 1990–2019: Update From the GBD 2019 Study. J Am Coll Cardiol. 2020;76(25):2982–3021. doi: 10.1016/j.jacc.2020.11.010 33309175 PMC7755038

[2] Okello S, Abeya FC, Lumori BAE, Akello SJ, Moore CC, Annex BH, et al. Validation of heart failure quality of life tool and usage to predict all-cause mortality in acute heart failure in Uganda: the Mbarara heart failure registry (MAHFER). BMC Cardiovasc Disord. 2018;18(1):232. doi: 10.1186/s12872-018-0959-1 30541443 PMC6291962

[3] Oyoo GO, Ogola EN. Clinical and socio demographic aspects of congestive heart failure patients at Kenyatta National Hospital, Nairobi. East Afr Med J. 1999;76(1):23–7. 10442143

[4] Aliku TO, Lubega S, Namuyonga J, Mwambu T, Oketcho M, Omagino JO, et al. Pediatric cardiovascular care in Uganda: Current status, challenges, and opportunities for the future. Ann Pediatr Cardiol. 2017;10(1):50–7. doi: 10.4103/0974-2069.197069 28163428 PMC5241845

[5] Kwan GF, Bukhman AK, Miller AC, Ngoga G, Mucumbitsi J, Bavuma C, et al. A simplified echocardiographic strategy for heart failure diagnosis and management within an integrated noncommunicable disease clinic at district hospital level for sub-Saharan Africa. JACC Heart Fail. 2013;1(3):230–6. doi: 10.1016/j.jchf.2013.03.006 24621875

[6] Carlson S, Duber HC, Achan J, Ikilezi G, Mokdad AH, Stergachis A, et al. Capacity for diagnosis and treatment of heart failure in sub-Saharan Africa. Heart. 2017;103(23):1874–9. doi: 10.1136/heartjnl-2016-310913 28490619

[7] Freers J, Mayanja-Kizza H, Ziegler JL, Rutakingirwa M. Echocardiographic diagnosis of heart disease in Uganda. Trop Doct. 1996;26(3):125–8. doi: 10.1177/004947559602600310 8783957

[8] Maro EE, Kaushik R. The role of echocardiography in the management of patients with congestive heart failure. "Tanzanian experience". Cent Afr J Med. 2009;55(5–8):35–9. doi: 10.4314/cajm.v55i5-8.63638 21977826

[9] Damasceno A, Cotter G, Dzudie A, Sliwa K, Mayosi BM. Heart failure in sub-saharan Africa: time for action. J Am Coll Cardiol. 2007;50(17):1688–93. doi: 10.1016/j.jacc.2007.07.030 17950152

[10] DeWyer A, Scheel A, Otim IO, Longenecker CT, Okello E, Ssinabulya I, et al. Improving the accuracy of heart failure diagnosis in low-resource settings through task sharing and decentralization. Glob Health Action. 2019;12(1):1684070. doi: 10.1080/16549716.2019.1684070 31694487 PMC6844369

[11] Ogedegbe G, Gyamfi J, Plange-Rhule J, Surkis A, Rosenthal DM, Airhihenbuwa C, et al. Task shifting interventions for cardiovascular risk reduction in low-income and middle-income countries: a systematic review of randomised controlled trials. BMJ Open. 2014;4(10):e005983. doi: 10.1136/bmjopen-2014-005983 25324324 PMC4202019

[12] Beaton A, Okello E, Scheel A, DeWyer A, Ssembatya R, Baaka O, et al. Impact of heart disease on maternal, fetal and neonatal outcomes in a low-resource setting. Heart. 2018. 10.1136/heartjnl-2018-313810 PMC11181686 30415203

[13] Longenecker CT, Kalra A, Okello E, Lwabi P, Omagino JO, Kityo C, et al. A Human-Centered Approach to CV Care: Infrastructure Development in Uganda. Glob Heart. 2018;13(4):347–54. doi: 10.1016/j.gheart.2018.02.002 29685638 PMC6258347

[14] Longenecker CT, Morris SR, Aliku TO, Beaton A, Costa MA, Kamya MR, et al. Rheumatic Heart Disease Treatment Cascade in Uganda. Circ Cardiovasc Qual Outcomes. 2017;10(11). doi: 10.1161/CIRCOUTCOMES.117.004037 29133472 PMC5728153

[15] Harris PA, Taylor R, Thielke R, Payne J, Gonzalez N, Conde JG. Research electronic data capture (REDCap)—a metadata-driven methodology and workflow process for providing translational research informatics support. J Biomed Inform. 2009;42(2):377–81. doi: 10.1016/j.jbi.2008.08.010 18929686 PMC2700030

[16] Ross AB, DeStigter KK, Rielly M, Souza S, Morey GE, Nelson M, et al. A low-cost ultrasound program leads to increased antenatal clinic visits and attended deliveries at a health care clinic in rural Uganda. PLoS One. 2013;8(10):e78450. doi: 10.1371/journal.pone.0078450 24205234 PMC3813603

[17] Langbecker D, Caffery LJ, Gillespie N, Smith AC. Using survey methods in telehealth research: A practical guide. J Telemed Telecare. 2017;23(9):770–9. doi: 10.1177/1357633X17721814 28728502

[18] Neergaard MA, Olesen F, Andersen RS, Sondergaard J. Qualitative description—the poor cousin of health research? BMC Med Res Methodol. 2009;9:52. doi: 10.1186/1471-2288-9-52 19607668 PMC2717117

[19] API XCD. Xe Currency Converter [Available from: https://www.xe.com/currencyconverter/.

[20] Kaplan RS, Anderson SR. Time-driven activity-based costing. Harv Bus Rev. 2004;82(11):131–8, 50. 15559451

[21] Chang AY, Nabbaale J, Nalubwama H, Okello E, Ssinabulya I, Longenecker CT, et al. Motivations of women in Uganda living with rheumatic heart disease: A mixed methods study of experiences in stigma, childbearing, anticoagulation, and contraception. PLoS One. 2018;13(3):e0194030. 10.1371/journal.pone.0194030 PMC5874006 29590159

[22] Frenk J, Chen L, Bhutta ZA, Cohen J, Crisp N, Evans T, et al. Health professionals for a new century: transforming education to strengthen health systems in an interdependent world. Lancet. 2010;376(9756):1923–58. doi: 10.1016/S0140-6736(10)61854-5 21112623

[23] Sani MU, Davison BA, Cotter G, Damasceno A, Mayosi BM, Ogah OS, et al. Echocardiographic predictors of outcome in acute heart failure patients in sub-Saharan Africa: insights from THESUS-HF. Cardiovasc J Afr. 2017;28(1):60–7. doi: 10.5830/CVJA-2016-070 28262911 PMC5514351

[24] Hoffman JI, Kaplan S. The incidence of congenital heart disease. Journal of the American College of Cardiology. 2002;39(12):1890–900. doi: 10.1016/s0735-1097(02)01886-7 12084585

[25] Hoffman J. The global burden of congenital heart disease. Cardiovasc J Afr. 2013;24(4):141–5. doi: 10.5830/CVJA-2013-028 24217047 PMC3721933

[26] Beaton A, Lu JC, Aliku T, Dean P, Gaur L, Weinberg J, et al. The utility of handheld echocardiography for early rheumatic heart disease diagnosis: a field study. European heart journal cardiovascular Imaging. 2015;16(5):475–82. 10.1093/ehjci/jeu296 PMC4542771 25564396

[27] Beaton A, Okello E, Lwabi P, Mondo C, McCarter R, Sable C. Echocardiography screening for rheumatic heart disease in Ugandan schoolchildren. Circulation. 2012;125(25):3127–32. doi: 10.1161/CIRCULATIONAHA.112.092312 22626741

[28] Okello E, Longenecker CT, Beaton A, Kamya MR, Lwabi P. Rheumatic heart disease in Uganda: predictors of morbidity and mortality one year after presentation. BMC Cardiovasc Disord. 2017;17(1):20. doi: 10.1186/s12872-016-0451-8 28061759 PMC5219796

[29] Okello E, Wanzhu Z, Musoke C, Twalib A, Kakande B, Lwabi P, et al. Cardiovascular complications in newly diagnosed rheumatic heart disease patients at Mulago Hospital, Uganda. Cardiovasc J Afr. 2013;24(3):80–5. doi: 10.5830/CVJA-2013-004 23736132 PMC3721959

[30] Ploutz M, Lu JC, Scheel J, Webb C, Ensing GJ, Aliku T, et al. Handheld echocardiographic screening for rheumatic heart disease by non-experts. Heart. 2016;102(1):35–9. doi: 10.1136/heartjnl-2015-308236 26438784

[31] Beaton A, Nascimento BR, Diamantino AC, Pereira GT, Lopes EL, Miri CO, et al. Efficacy of a Standardized Computer-Based Training Curriculum to Teach Echocardiographic Identification of Rheumatic Heart Disease to Nonexpert Users. The American journal of cardiology. 2016;117(11):1783–9. doi: 10.1016/j.amjcard.2016.03.006 27084054

[32] Diamantino A, Beaton A, Aliku T, Oliveira K, Oliveira C, Xavier L, et al. A focussed single-view hand-held echocardiography protocol for the detection of rheumatic heart disease. Cardiol Young. 2017:1–10. 10.1017/S1047951117001676 28889812

[33] Webb CL, Waugh CL, Grigsby J, Busenbark D, Berdusis K, Sahn DJ, et al. Impact of telemedicine on hospital transport, length of stay, and medical outcomes in infants with suspected heart disease: a multicenter study. Journal of the American Society of Echocardiography: official publication of the American Society of Echocardiography. 2013;26(9):1090–8. 23860093 10.1016/j.echo.2013.05.018

[34] Sable CA, Cummings SD, Pearson GD, Schratz LM, Cross RC, Quivers ES, et al. Impact of telemedicine on the practice of pediatric cardiology in community hospitals. Pediatrics. 2002;109(1):E3. doi: 10.1542/peds.109.1.e3 11773571

[35] Finley JP, Sharratt GP, Nanton MA, Chen RP, Bryan P, Wolstenholme J, et al. Paediatric echocardiography by telemedicine—nine years’ experience. Journal of telemedicine and telecare. 1997;3(4):200–4. doi: 10.1258/1357633971931165 9614734

[36] Fisher JB, Alboliras ET, Berdusis K, Webb CL. Rapid identification of congenital heart disease by transmission of echocardiograms. American heart journal. 1996;131(6):1225–7. doi: 10.1016/s0002-8703(96)90103-9 8644607

[37] Sable C, Roca T, Gold J, Gutierrez A, Gulotta E, Culpepper W. Live transmission of neonatal echocardiograms from underserved areas: accuracy, patient care, and cost. Telemedicine journal: the official journal of the American Telemedicine Association. 1999;5(4):339–47. 10908449 10.1089/107830299311907

[38] Sobczyk WL, Solinger RE, Rees AH, Elbl F. Transtelephonic echocardiography: successful use in a tertiary pediatric referral center. The Journal of pediatrics. 1993;122(6):S84–8. doi: 10.1016/s0022-3476(09)90049-x 8501554

[39] Lewin M, Xu C, Jordan M, Borchers H, Ayton C, Wilbert D, et al. Accuracy of paediatric echocardiographic transmission via telemedicine. Journal of telemedicine and telecare. 2006;12(8):416–21. doi: 10.1258/135763306779378636 17227608

[40] Houston A, McLeod K, Richens T, Doig W, Lilley S, Murtagh E, et al. Assessment of the quality of neonatal echocardiographic images transmitted by ISDN telephone lines. Heart. 1999;82(2):222–5. doi: 10.1136/hrt.82.2.222 10409540 PMC1729137

[41] Scholz TD, Kienzle MG. Optimizing utilization of pediatric echocardiography and implications for telemedicine. The American journal of cardiology. 1999;83(12):1645–8. doi: 10.1016/s0002-9149(99)00171-x 10392869

[42] McCrossan BA, Grant B, Morgan GJ, Sands AJ, Craig B, Casey FA. Diagnosis of congenital heart disease in neonates by videoconferencing: an eight-year experience. Journal of telemedicine and telecare. 2008;14(3):137–40. doi: 10.1258/jtt.2008.003011 18430281

[43] Awadallah S, Halaweish I, Kutayli F. Tele-echocardiography in neonates: utility and benefits in South Dakota primary care hospitals. South Dakota medicine: the journal of the South Dakota State Medical Association. 2006;59(3):97–100. 16566300

[44] Krishnan A, Fuska M, Dixon R, Sable CA. The evolution of pediatric tele-echocardiography: 15-year experience of over 10,000 transmissions. Telemedicine journal and e-health: the official journal of the American Telemedicine Association. 2014;20(8):681–6. doi: 10.1089/tmj.2013.0279 24841367

[45] Satou GM, Rheuban K, Alverson D, Lewin M, Mahnke C, Marcin J, et al. Telemedicine in Pediatric Cardiology: A Scientific Statement From the American Heart Association. Circulation. 2017. doi: 10.1161/CIR.0000000000000478 28193604

[46] Cavero E, Alesanco A, Castro L, Montoya J, Lacambra I, Garcia J. SPIHT-based echocardiogram compression: clinical evaluation and recommendations of use. IEEE J Biomed Health Inform. 2013;17(1):103–12. doi: 10.1109/TITB.2012.2227336 23193314

[47] LaGrone LN, Sadasivam V, Kushner AL, Groen RS. A review of training opportunities for ultrasonography in low and middle income countries. Trop Med Int Health. 2012;17(7):808–19. doi: 10.1111/j.1365-3156.2012.03014.x 22642892

[48] Choi BG, Mukherjee M, Dala P, Young HA, Tracy CM, Katz RJ, et al. Interpretation of remotely downloaded pocket-size cardiac ultrasound images on a web-enabled smartphone: validation against workstation evaluation. Journal of the American Society of Echocardiography: official publication of the American Society of Echocardiography. 2011;24(12):1325–30. doi: 10.1016/j.echo.2011.08.007 21925836

[49] Ross AB, DeStigter KK, Coutinho A, Souza S, Mwatha A, Matovu A, et al. Ancillary benefits of antenatal ultrasound: an association between the introduction of a low-cost ultrasound program and an increase in the numbers of women receiving recommended antenatal treatments. BMC Pregnancy Childbirth. 2014;14:424. doi: 10.1186/s12884-014-0424-9 25522741 PMC4296687

[50] Bagayoko CO, Traore D, Thevoz L, Diabate S, Pecoul D, Niang M, et al. Medical and economic benefits of telehealth in low- and middle-income countries: results of a study in four district hospitals in Mali. BMC Health Serv Res. 2014;14 Suppl 1:S9. doi: 10.1186/1472-6963-14-S1-S9 25080312 PMC4108933

[51] Bhavnani SP, Sola S, Adams D, Venkateshvaran A, Dash PK, Sengupta PP, et al. A Randomized Trial of Pocket-Echocardiography Integrated Mobile Health Device Assessments in Modern Structural Heart Disease Clinics. JACC Cardiovasc Imaging. 2017. doi: 10.1016/j.jcmg.2017.06.019 28917688

[52] Cannon J, Roberts K, Milne C, Carapetis JR. Rheumatic Heart Disease Severity, Progression and Outcomes: A Multi-State Model. J Am Heart Assoc. 2017;6(3). doi: 10.1161/JAHA.116.003498 28255075 PMC5523987

[53] Larsen-Cooper E, Bancroft E, Rajagopal S, O’Toole M, Levin A. Scale Matters: A Cost-Outcome Analysis of an m-Health Intervention in Malawi. Telemedicine journal and e-health: the official journal of the American Telemedicine Association. 2016;22(4):317–24. doi: 10.1089/tmj.2015.0060 26348994 PMC4817568

[54] Roberts K, Cannon J, Atkinson D, Brown A, Maguire G, Remenyi B, et al. Echocardiographic Screening for Rheumatic Heart Disease in Indigenous Australian Children: A Cost-Utility Analysis. J Am Heart Assoc. 2017;6(3). doi: 10.1161/JAHA.116.004515 28255077 PMC5524001

[55] Watkins D, Lubinga SJ, Mayosi B, Babigumira JB. A Cost-Effectiveness Tool to Guide the Prioritization of Interventions for Rheumatic Fever and Rheumatic Heart Disease Control in African Nations. PLoS Negl Trop Dis. 2016;10(8):e0004860. doi: 10.1371/journal.pntd.0004860 27512994 PMC4981376

[56] Lopes EL, Beaton AZ, Nascimento BR, Tompsett A, Dos Santos JP, Perlman L, et al. Telehealth solutions to enable global collaboration in rheumatic heart disease screening. Journal of telemedicine and telecare. 2016. doi: 10.1177/1357633X16677902 27815494

[57] Eberly LA, Rusangwa C, Ng’ang’a L, Neal CC, Mukundiyukuri JP, Mpanusingo E, et al. Cost of integrated chronic care for severe non-communicable diseases at district hospitals in rural Rwanda. BMJ Glob Health. 2019;4(3):e001449. doi: 10.1136/bmjgh-2019-001449 31321086 PMC6597643

