# Supplementary material for: Establishment of a cardiac telehealth program to support cardiovascular diagnosis and care in a remote, resource-poor setting in Uganda
Source: PLoS One. 2021 Aug 6;16(8):e0255918. doi: 10.1371/journal.pone.0255918 (PMC8345851; doi:10.1371/journal.pone.0255918)
Supplement: S1 File — (PDF) [file pone.0255918.s001.pdf]

Appendix 1: Clinical REDCap instruments

Gulu Intake Form

Record ID	____________________
Consent signed	◯ Yes<br>◯ No
Location:	◯ OPD<br>◯ Inpatient
Age Group:	◯ Pediatric<br>◯ Adult
Date:	____________________
Type of Visit:	◯ New<br>◯ Follow-up
**Demographics**	
Surname:	____________________
First Name:	____________________
Village:	____________________
Parish:	____________________
District:	____________________
Subcounty:	____________________
Telephone Number:	____________________
Telephone Number #2:	____________________
Telephone Number #3:	____________________
Age:	____________________
Sex:	◯ Male<br>◯ Female

Distance Traveled from home to clinic (patient reports)	______ ((km))
Calculated distance traveled from home to clinic (performed by GRRH staff)	______ ((km))
Amount of travel time from home to clinic (hours)	______
Additional travel costs: meals	______
Additional travel costs: accommodation	______
Additional travel costs: transport	______
Job/source of income	______
Salary (monthly household income)	◯ 0 - 50,000 UGX<br>◯ 50 - 100,000 UGX<br>◯ 000 - 150,000 UGX<br>◯ Greater than 150,000 UGX
Days of school/work missed:	______
Alternative Care to Telemedicine:	◯ No care<br>◯ Wait for next Twalib clinic<br>◯ Travel to Kampala
Weight:	______ ((kg))
Height:	______ ((cm))
Heart Rate:	______ ((bpm))
SpO2:	______ ((%))
Blood Pressure:	______ (mmHg)

Patient Complaints

	Yes	No
Chest pain	○	○
Shortness of breath	○	○
Leg edema	○	○
Abdominal swelling	○	○
Fainting	○	○
Palpitations	○	○
Decreased exercise capacity	○	○
failure to thrive (pediatrics)	○	○
joint pain/swelling (pediatrics)	○	○

Other patient complaints: ____________________

Past Medical History

	Yes	No
Hypertension	○	○
ARF/RHD	○	○
Stroke	○	○
Valve leakage/stenosis	○	○
Arrhythmia	○	○
Cardiomyopathy	○	○
Congenital heart defect	○	○
Diabetes	○	○
Kidney disease	○	○
Lung disease	○	○

Other past medical history: ____________________

Current Medications

	Yes	No
Lasix	○	○
Digoxin	○	○
Captopril/Enalapril	○	○
Propranolol	○	○
Warfarin	○	○

Other Medications: ____________________

Physical Exam

	Yes	No
Cyanosis/Clubbing	○	○
Decreased lung sounds	○	○
Irregular heart rhythm	○	○
Murmur	○	○
Abdominal swelling	○	○
Leg edema	○	○

Other findings on physical exam: ______________________

Tests

	Yes	No
ECG completed	○	○
Echo completed	○	○

ECG Upload

(picture)

UHI Consultation Form

Date: ______________________

Name of Provider Reviewing: ______________________

ECG

Rhythm: ______________________

Other Findings: ______________________

Summary: ______________________

ECHO

Atrioventricular valves morphology/regurgitation/stenosis: ______________________

Semilunar valve morphology/regurgitation/stenosis: ______________________

Chamber size/thickness: ______________________

Ventricular function: ______________________

Wall motion: ______________________

Pericardium: ______________________

Congenital heart defects: ______________________

Other: ______________________

Echo summary: ______________________

Diagnoses

Diagnoses	☐ Normal<br>☐ Hypertensive Heart Disease<br>☐ Valvular/Rheumatic Heart Disease<br>☐ Dilated cardiomyopathy/ischemia<br>☐ Congenital heart disease<br>☐ Isolated right sided heart disease<br>☐ Pericardial disease<br>☐ Arrhythmia
Other Diagnoses:	____________________
Medications (name, dose, duration)	________________________
Other recommendations:	________________________
Follow up	◯ None<br>◯ Telemedicine follow up<br>◯ Outpatient follow up (Twalib clinic visit)<br>◯ Admission GRRH<br>◯ Transfer to UHI
Follow up date (if needed):	____________________
