# Supplementary material for: Establishment of a cardiac telehealth program to support cardiovascular diagnosis and care in a remote, resource-poor setting in Uganda
Source: PLoS One. 2021 Aug 6;16(8):e0255918. doi: 10.1371/journal.pone.0255918 (PMC8345851; doi:10.1371/journal.pone.0255918)
Supplement: S2 File — (DOCX) [file pone.0255918.s002.docx]

**Appendix 2: Echocardiography Protocol**

1. **Parasternal long axis view**
   1. **2D: Loop 1**
      1. Left ventricular function
      2. Septal thickness
      3. Mitral valve morphology and stenosis
      4. Pericardial effusion
      5. Aortic valve morphology
   2. **Color Doppler**
      1. Mitral valve inflow/regurgitation: Loop 2 (If MR present, freeze and measure MR jet, store frame – **Still 1**)
      2. Aortic valve outflow/ regurgitation: Loop 3 (if AR present, freeze and measure AR jet, store frame – **Still 2**)
2. **Parasternal short axis:**
   1. Left ventricle: Loop 4 (at level of left ventricle papillary muscles)
      1. Left ventricular function
      2. Regional wall motion abnormalities/Septal motion
      3. Pericardial effusion
   2. Mitral valve morphology: Loop 5
   3. Mitral valve color: Loop 6
   4. Aortic valve: morphology: Loop 7
   5. Aortic valve color: Loop 8
   6. Pulmonary valve and branches morphology: Loop 9
   7. Pulmonary valve and branches color: Loop 10
3. **Apical four/five chamber view**
   1. **2D**
      1. Left ventricular function, effusion, and size: Loop 11
      2. Right ventricular function, effusion, and size: Loop 12
      3. Aortic valve: Loop 13
   2. **Color Doppler**
      1. Mitral inflow/regurgitation: Loop 14 (If MR present, freeze and measure MR jet, store frame – **Still 3**)
      2. Tricuspid inflow/regurgitation: Loop 15
      3. Aortic valve outflow/regurgitation: Loop 16 (if AR present, freeze and measure AR jet, store frame – **Still 4**)
4. **Subcostal views**
   1. **Four chamber/3 O’clock view (for all children 17 and under and adults when possible)**
      1. Atrial septum/mitral inflow morphology: Loop 17
      2. Atrial septum/mitral inflow color (atrial septum): Loop 18
      3. Left ventricular outflow morphology: Loop 19
      4. Left ventricular outflow color: Loop 20
      5. Right ventricular outflow morphology: Loop 21
      6. Right ventricular outflow color: Loop 22
   2. **Short axis/6 O’clock view**
      1. IVC: Loop 23 (Freeze and measure IVC diameter, store frame – **Still 5)**
