# Supplementary material for: Establishment of a cardiac telehealth program to support cardiovascular diagnosis and care in a remote, resource-poor setting in Uganda
Source: PLoS One. 2021 Aug 6;16(8):e0255918. doi: 10.1371/journal.pone.0255918 (PMC8345851; doi:10.1371/journal.pone.0255918)
Supplement: S3 File — (DOCX) [file pone.0255918.s003.docx]

**Appendix 3: Thematic Coded Responses to Focus Group Discussions**

| **Theme** | **Exemplifying Quotes** |
| --- | --- |
| **Benefit of telemedicine** | …I was to go to Mulago it would be too costly in terms of transport and other additional costs. As peasant farmers we barely have that money they may need and sometimes it may lead to death since you can afford to pay for the medication. Now that everything is done from here has really helped us and the cost is not compared to going to Mulago.  ..from here to Mulago and from Mulago it may again take 2 weeks but from here [GRRH] the result only take 2 or 3 days.  This machine has help in a way that sometimes as a mother it may not be easy to travel when you have a young child at home and also the transport cost. But from here after finishing the scan you just wait for the result to come so it has helped me a lot.  We discuss about telemedicine and to me I think this machine is good because I couldn’t go up to Mulago, for some of us here going to Mulago may not be so easy because of the transport cost, secondly when you reach there you may even get lost since you are not familiar with the place, accommodation, food etc. so I think we are just blessed to have everything here in Gulu, you just come from home and after finishing everything you go back home which is very easy. |
| **Acceptance and gratitude** | This service has helped us a lot because without this machine I would not know that my child is having a heart problem.  When I come here, they explain to me everything and I was meditating from my mind that it’s just like talking on phone whereby someone can be at very far distance still you are able to share words. So, I have hope about telemedicine and I know that it works well and I was happy when my results came back, I had no doubts about it as well. |
| **Belief in system** | I wish this service could reach other nearing districts like Nwoya, Pakwach, Arua so that it helps other people as well because a lot of people are dying because of lack of information about this disease.  .  What I would like to say is that if the government could add more of this machine to enable easy work. And another thing is that they should train more people to use this machine.  And also, I have a request to the Government if they could continue with this program and extend it to health centers so that even the rural people also benefits. |
| **Understanding of telemedicine system** | So, when I find out that my child was having complications, he said I should come here (GRRH) because the machine they use is called telemedicine. The diagnosis and test results are sent via internet to Mulago then from there they prescribe the medicine to the problem and later they call you on phone to come and pick the results.  When I came here for the scan, they said they will scan me from here and it will be sent to Kampala via internet and from there they will work on it and then send the result back. |
| **Disadvantages/limitations** | I had hope that it’s going to be different and there will be no doubt or mistake since machines are more accurate than human beings.  Now that my child has been diagnosed with this heart condition, how will I know that her health is improving or not? Because they are not giving any medication but every time, they call me to come here they just do other things and there’s nothing like medication or something serious they tell me. they always tell me to wait for the phone calls.  I also request the hospital to add more nurses here to make work easy and help follow the client and monitor them closely how they are improving.  ,… this length of time of which they could work on you they first send this thing to Mulago and they download, and then send back the result and like for a whole day is a risk. Because in the process of doing all that the person can loss his or her life as well  I think the time duration of producing the results from Mulago shouldn’t be too wide. At least if they could reduce it to 4 or 5 hours it would be okay |
| **Doctor-patient contact still highly valued** | One bad thing about telemedicine is that, you do not have a physical interaction with your doctor to explain more of what you feel or how you feel. And secondly using telemedicine may lose hope since you are not seeing the doctor directly so how can I believe it,…”  So, what am trying to appeal to the government is that telemedicine is a good thing and I believe it will help Ugandans if maybe they try to induce other policy whereby they elevate specialists who works on the heart such that they could be in each and every big hospitals in the country, it will help a lot.  I advise the government if possible, they should at least try to bring for us some doctors that can be based here at GRRH or other regional referral hospitals in Uganda so that we also have interactions with them though we know telemedicine is working well but once you interact with them directly it will be very great.  I advise the government to improve on this system and also let the doctors come here and explain to the patients because most of us have challenges and don’t understand or believe this things so if they could come at least twice a month so that we interact with them physically.  , …they should encourage follow-up for these patients who have been confirm having this disease so that when follow-up is done like from home, it also makes the patient feel loved, encouraged and cared for. And sometimes he/she will have that confidence of getting well again and it also relieves the pains someone is having in the heart. So I advise them to do follow-ups if possible and provide them with the services needed. |
| **May not work in an emergency** | To me that I didn’t like is that, the scan is done from here and they tell you to wait. Meaning a patient could even lose his/her life, am also requesting if you start working on someone its better you finish everything at once without any delay other than waiting for the result from a distant and start giving medicines. So that’s the reason why I think it’s not so good and I feel if everything is done at once it also saves the patients.  Indeed, when I came here, they were trying to examine me with this telemedicine, it has been perfect although the challenge I notice with this system is that it is too durable because if you look at the duration from the time they do scan, the transfer to Mulago online and someone download to work on it, sometimes when your condition is bad would not help you in time. |
| **Technical limitations** | The machine uses electricity so when there’s no power it affects its efficiency.  The bad side of using this machine is that it uses electricity so when power go off in the process it may also affect the results. |
| **Lengthy wait time** | I think the bad side of using telemedicine is that in case you are desperate and you want to know the result at that very time it’s impossible, it takes a few days for the result to reach you and it stresses you because you keep thinking about it.  I think if possible that very day they do scan, they should give out the result at least within an hour so that it let the patient and the caregiver also go back home feeling relieve and less worried. |
